# The Pressor Response to the Drinking of Cold Water and Cold Carbonated Water in Healthy Younger and Older Adults

**DOI:** 10.3389/fneur.2021.788954

**Published:** 2022-01-10

**Authors:** Satoshi Kubota, Yutaka Endo, Mitsue Kubota, Hiroko Miyazaki, Tomohiko Shigemasa

**Affiliations:** ^1^School of Health Sciences at Odawara, International University of Health and Welfare, Odawara, Japan; ^2^Graduate School, Japanese Red Cross College of Nursing, Tokyo, Japan; ^3^Department of Cardiology, Yokohama Brain and Spine Center, Yokohama, Japan

**Keywords:** pressor response, cold water, cold carbonated water, drink, age

## Abstract

**Purpose:** Water drinking has been proposed for the treatment of orthostatic hypotension because it can increase blood pressure in patients. This study aimed to investigate whether drinking water with a cold or carbonation stimulus would cause a more effective pressor response, and whether it would be greater in older than in younger adults.

**Methods:** We assessed blood pressure and heart rate from non-invasive arterial pressure (a volume-clamp method) and type II electrocardiography in 13 healthy young adults (6 females, 7 males; mean age, 19.9 ± 1.1 years) and nine healthy older adults (all females; mean age, 71.4 ± 4.2 years) who drank 200 mL of cold, cold carbonated, and room temperature water.

**Results:** The pressor response to the drinking of cold and cold carbonated water was greater than that to room temperature water in both younger and older participants (*p* < 0.05; changes in systolic blood pressure of room temperature water, cold water and cold carbonated water in young: 15.31 ± 9.66, 22.56 ± 11.51 and 32.6 ± 17.98 mmHg, respectively; changes in systolic blood pressure of room temperature water, cold water and cold carbonated water in elderly: 21.84 ± 14.31, 41.53 ± 19.82 and 48.16 ± 16.77 mmHg, respectively). In addition, the pressor response to cold and cold carbonated water was persistent during the recovery period by about 5–10 mmHg (*p* < 0.05). Furthermore, the pressor response during the drinking and recovery periods was greater in the older than in the younger participants (*p* < 0.05).

**Conclusion:** Our data suggest that even smaller amounts of water are able to elicit a sustained pressor response, in particular if the water is cold and carbonated. We speculate that the pressor effect may render cold and carbonated water an appropriate first aid method against certain forms of acute hypotension.

## Introduction

Orthostatic hypotension (OH) is reported to be a risk factor for falls and mortality (1), making the development of methods to prevent OH a clinically critical challenge. OH is caused by the shift of blood to lower parts of the body during orthostasis and the consequent lack of proper blood pressure regulation (1, 2). The potential risk of OH is especially high in the elderly patients, who may develop it just by sitting after 12 h or more of bed rest (3). In the treatment of OH, in addition to pharmacological treatment, bandaging, and posture adjustment, the method of water drinking has been proposed (2, 4).

It is well known that elevated blood pressure occurs after water drinking in older patients and those with autonomic failure (5–7). Although the mechanism of the pressor response to water might be the effect of osmotic changes, the concrete mechanism remains unclear (1, 8). Drinking 500 mL of water at a time is recommended for the treatment of OH (2, 7, 9). However, it is not easy for frail patients to drink this amount of water at once, we believe that a smaller amount would be more applicable.

Endo et al. (10) showed that mean blood pressure (MBP) increased by about 10 mmHg during drinking and then rapidly disappeared. This pressor response is instantaneous and differs from the response due to hypoosmolality after water ingestion as other studies have addressed (11), it has been suggested to be induced by afferent signals of muscle mechanoreceptors related to water drinking and sympathetic efferent nerves (12, 13). If a small amount of water can be used to induce the pressor response, it may be an effective first aid intervention to improve acute hypotensive symptoms. It is also known that the nociceptive stimulus causes an increase in blood pressure (14). While drinking water, adding nociceptive stimulus such as carbonation (15) or cold (16) to the drinking water may increase the pressor response. Furthermore, the pressor response may be greater in the elderly than in the young due to age-related decreases in autonomic function and arterial compliance.

In the present study, we investigated the effect of drinking a small amount of water with a cold or carbonation stimulus intervention on blood pressure and compared elderly and young adults. We hypothesized that drinking even a small amount of cold water or cold carbonated water would cause a more effective pressor response, which would be especially pronounced in the elderly, and that the pressor response would disappear immediately after drinking the water.

## Methods

We determined non-invasive arterial pressure and heart rate (HR) in healthy younger and older adults who drank 200 mL of cold, cold carbonated, and room temperature water. We also calculated cardiovagal baroreflex sensitivity (BRS) and respiratory sinus arrhythmia (RSA) from arterial pressure and HR to compare autonomic cardiovascular regulatory function in the younger and older participants. To investigate the effect of drinking water on body temperature, we determined core body temperature using the same protocol as in the hemodynamic experiments. All results are presented as the mean ± SD.

### Participants

The participants in the hemodynamic experiments were 13 healthy young adults (6 females and 7 males; 19.9 ± 1.1 years; height, 168.1 ± 5.4 cm; weight, 59.0 ± 6.8 kg) and nine healthy older adults (all females; age, 71.4 ± 4.2 years; height, 152.4 ± 3.9 cm; weight, 48.6 ± 4.6 kg). This sample size was determined with reference to the study by Endo et al. (10) on the pressor response during the drinking water. The participants of the experiments measuring core temperature were 12 healthy young adults (4 males and 8 females; age, 21.08 ± 0.67 years; height, 165.58 ± 8.77 cm; weight, 56.50 ± 9.7 kg) and 10 healthy older adults (all females; age, 72 ± 4.3 years; height, 152.45 ± 3.7 cm; weight, 49.05 ± 4.6 kg). None of the participants had a history of internal or cardiovascular disease. All of the participants took part in an interview, underwent blood pressure and electrocardiogram (ECG) measurements, and were confirmed as being healthy by the physician in charge and researcher before participating in the experiment. Participants wore light clothing, with male participants wearing only a shorts and female participants wearing a short sleeve top and shorts. Experiments in young females proceeded excluding menstruation of the menstrual cycle.

All of the participants were asked to avoid consuming caffeine and alcohol and engaging in strenuous exercise from the day before the experiment, and to refrain from drinking anything other than water after dinner. The participants were also asked to abstain from food and drinks for 2 hours before the experiment on the experimental day.

This study was approved by the Ethics Committee of the International University of Health and Welfare (17-Io-73). Written informed consent was obtained from all participants after they were provided with adequate information about the study protocol, procedures, and risks.

### Protocol

All experiments proceeded between 10:30 and 15:00. The room temperature was adjusted to thermoneutral temperature (about 28°C). The measurement protocol consisted of 10 min of control, 1 min of drinking 200 mL of water, and 20 min of recovery. During the measurements, the participants remained in a resting seated position. The water used for drinking was room temperature mineral water (Oishii Mizu; Asahi, Tokyo, Japan) at about 28°C, cold mineral water (Oishii Mizu; Asahi) at about 4°C, or cold carbonated water (Wilkinson; Asahi) at about 4°C. To drink water at a constant rate per minute, the participants drank the water supplied by the investigator using a syringe. Each participant consumed each type of water on different days in randomized order. We generated random numbers from a uniform distribution and determined their order using the statistical programming language R (R Core Team, 2020; version 4.0.3). All participants participated in the experiment with a washout period of at least one day.

### Measurements

Arterial pressure was measured non-invasively using a volume-clamp method (Portapress; Finapres Medical Systems, Enschede, the Netherlands). A cuff was attached to the right index finger and adjusted so that it would be maintained at heart level. HR was calculated using a type II ECG (ECG 100C; BIOPAC Systems, Santa Barbara, CA, USA). We measured the tympanic membrane temperature as the core temperature non-invasively using an earphone-type infrared tympanic thermometer (CE Thermo; Nipro, Osaka, Japan) Kiya et al. (17). The probe of the thermometer was inserted into the ear canal for measurement.

Arterial pressure was recorded with ECG by outputting the pressure pulse wave as an analog signal from the Portapress. The data of all experiments were stored on a hard disk at a sampling rate of 1,000 Hz through an analog-to- digital converter (MP-150; BIOPAC Systems).

### Data Analysis and Statistics

We calculated Systolic blood pressure (SBP), MBP, and diastolic blood pressure (DBP) per beat from the measured pressure pulse wave, and HR and RR interval (RRI) from the ECG using AcqKnowledge 5.0 (BIOPAC Systems, Santa Barbara, CA, USA), a data acquisition and analysis software program. We checked that the blood pressure, HR, and core temperature during the control period were steady and without extreme changes and analyzed them.

RSA, an index of vagal modulation (18, 19), was calculated from the power spectrum of the RR interval (0.15–0.4 Hz) using the maximum entropy method with high resolution (20). Time series data of RR intervals with non-stationary can affect the results of spectral analysis (21, 22). Therefore, we checked the time series data for outliers prior to the spectral analysis and confirmed that they were stationary. The BRS was calculated from the first 5 min of the control periods' RR interval and SBP using the sequence technique (23, 24). The beat-to-beat time series of RRI and SBP were scanned to identify sequences in which RRI and SBP increased or decreased for three or more consecutive beats. In addition, the minimum change threshold was 1 mmHg for SBP and 4 ms for RRI. Linear regression with correlation coefficients >0.85 for sequences was analyzed. The means of the individual slopes of all SBP-RRI sequences were calculated as BRS. The BRS reflects vagally mediated cardiac baroreflex responses (25, 26). BRS was calculated using Nevrokard BRS software (Nevrokard, Izola, Slovenia). RSA and BRS were log-transformed in accordance with a previous study (27, 28).

We calculated the change in blood pressure and HR during the drinking and recovery periods from the first 5 min of the control period, and the mean values were calculated for 1 min of the drinking periods and 5-min of the recovery period (0–5, 5–10, 10–15, and 15–20 min). The statistical analyses took repeated measures into account, and we used a linear mixed model (LMM) to examine the effects of water type and time course on blood pressure and HR for each generation (older and younger). Next, we constructed a model with water samples, periods, and interactions as fixed effects and the participants as random intercepts, and then calculated f-values and degrees of freedom using the Kenward–Roger method. When an interaction was observed, the effect was evaluated for each factor using the LMM. The Holm method was applied (to adjust *p*-values) for multiple comparisons. Welch's *t*-test was performed to compare the younger and older participants. We also calculated cohen's d (d) as the effect size. For the results of the comparison of the two groups with significant differences, *p*-values and cohen's d are shown as *p*-value (cohen's d).

The core temperature in the recovery period was calculated as the change from 10 min in the control period, and the average value was calculated for every 10 min of the recovery period (0–10 and 10–20 min). To investigate the effects of the temperature of water samples on core temperature, the LMM was used as in the hemodynamic experiments.

We used R (R Core Team, 2020; version 4.0.3) and the lme4 package, emmeans package, pbkrtest package, and effsize package for the statistical analysis. The level of statistical significance was set at <5%.

## Results

### Hemodynamics

Table 1 shows the blood pressure and HR values in the control period. In the LMM with water samples as fixed effects and participants as random intercepts, there was no main effect of water type on values (SBP, *p* = 0.9323; MBP, *p* = 0.9169; DBP, *p* = 0.6763; HR, *p* = 0.1007). In addition, no significant differences in blood pressure and HR were seen between the younger and older participants using Welch's *t*-test (SBP of room water, cold water and cold carbonated water: *p* = 0.6606, *p* = 0.3304 and *p* = 0.2326, respectively; MBP of room water, cold water and cold carbonated water: *p* = 0.6438, *p* = 0.7182 and *p* = 0.5757, respectively; DBP of room water, cold water and cold carbonated water: *p* = 0.252, *p* = 0.7648 and *p* = 0.8762, respectively; HR of room water, cold water and cold carbonated water: *p* = 0.5204, *p* = 0.7283 and *p* = 0.4677, respectively).

**Table 1:** Blood pressure and heart rate at control period by water type in experiment 1.

**Age**	**Water**	**SBP (mmHg)**			**MBP (mmHg)**			**DBP (mmHg)**			**HR (bpm)**		
		**Mean**	**SD**		**Mean**	**SD**		**Mean**	**SD**		**Mean**	**SD**	
Younger (*n* = 13) n.m.	Room	102.11	18.81	n.s.	72.82	13.7	n.s.	58.17	11.48	n.s.	78.57	12.53	n.s.
	Cold	100.34	12.74	n.s.	70.58	9.06	n.s.	55.7	7.72	n.s.	75.87	9.13	n.s.
	CO_2_	99.05	13.88	n.s.	71.18	9.98	n.s.	57.25	8.73	n.s.	82	8.43	n.s.
Older (*n* = 9) n.m.	Room	106.06	21.34		70.15	12.58		52.2	11.69		75.63	8.59	
	Cold	107.46	18.27		72.23	11.1		54.61	8.6		77.53	11.83	
	CO_2_	107.7	17.38		73.68	10.17		56.66	8.35		78.91	10.26	

*n.m. No main effect of water type (p > 0.05)*.

*n.s. No significant differences (p > 0.05) vs. older adults*.

*SBP, systolic blood pressure; MBP, mean blood pressure; DBP, diastolic blood pressure; HR, heart rate; SD, standard deviation*.

**Figure 1:**
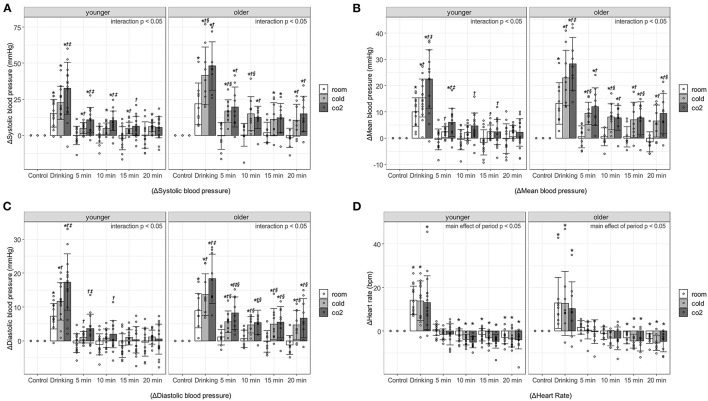
Changes in blood pressure and heart rate against control period, associated with age, water type, and period. **(A)** Systolic blood pressure. **(B)** Mean blood pressure. **(C)** Diastolic blood pressure. **(D)** Heart rate. Values are shown as mean ± standard deviation, ^*^p < 0.05 vs. control period, †*p* < 0.05 vs. room, ‡ *p* < 0.05 vs. cold, § *p* < 0.05 vs. younger. 5 min, recovery 0-5min; 10 min, recovery 5-10min; 15 min, recovery 10-15min; 20 min, recovery 15-20min. Room, room temperature water; Cold, cold water; CO_2_, cold carbonated water.

Figure 1 shows the blood pressure and HR responses to water drinking in each period. Interaction effects were observed for change in SBP (ΔSBP), change in MBP (ΔMBP), and change in DBP (ΔDBP) in the younger and older participants (*p* < 0.0001), so multiple comparisons were performed after evaluating the effects of each factor separately. On the other hand, regarding change in HR (ΔHR), no interaction (younger, *p* = 0.951; older, *p* = 0.9975) or main effect of water type (younger, *p* = 0.0555; older, *p* = 0.3507) was observed in the younger or older participants; only a main effect of period (younger, *p* < 0.0001; older, *p* < 0.0001) was observed.

Blood pressure increased significantly during the 1-min drinking period. In particular, ΔSBP during the drinking period of cold water and cold carbonated water was over 40 mmHg in the older adults [room water, 21.84 ± 14.31 mmHg, *p* < 0.000(2.81); cold water, 41.53 ± 19.82 mmHg, *p* < 0.0001(4.21); cold carbonated water, 48.16 ± 16.77 mmHg, *p* < 0.0001(5.5)], and about 20–30 mmHg in the young [room water, 15.31 ± 9.66 mmHg, *p* < 0.0001(2.68); cold water, 22.56 ± 11.51 mmHg, *p* < 0.0001(4.43); cold carbonated water, 32.6 ± 17.98 mmHg, *p* < 0.0001(4.19)]. In the younger and older participants, the pressor responses to cold and cold carbonated water were significantly higher than that to room temperature water during the drinking period [ΔSBP of cold water in older adults: *p* = 0.0021(1.884); ΔSBP of cold carbonated water in younger adults: *p* = 0.0006(1.729); ΔSBP of cold carbonated water in older adults: *p* = 0.0002 (1.884); ΔMBP of cold water in younger adults: *p* = 0.0497(0.778); ΔMBP of cold water in older adults: *p* = 0.0017(1.93); ΔMBP of cold carbonated water in younger adults: *p* = 0.0002(1.791); ΔMBP of cold carbonated water in older adults: *p* < 0.0001(2.99); ΔDBP of cold water in younger adults: *p* = 0.0287(0.876); ΔDBP of cold water in older adults: *p* = 0.0119(1.49); ΔDBP of cold carbonated water in younger adults: *p* < 0.0001(1.971); ΔDBP of cold carbonated water in older adults: *p* < 0.0001(2.96)]. Furthermore, in both groups, the pressor response to cold carbonated water was significantly higher than that to cold water during the drinking period [ΔSBP in younger adults: *p* = 0.0344(1.004); ΔMBP in younger adults: *p* = 0.0252(1.013); ΔMBP in older adults: *p* = 0.0392(1.06); ΔDBP in younger adults: *p* = 0.015(1.096); ΔDBP in older adults: *p* = 0.0119(1.47)].

In younger participants during the recovery period, ΔSBP was markedly increased by cold and cold carbonated water. Cold carbonated water significantly increased SBP at 0–5 and 5–10 min of the recovery period [0–5min, *p* = 0.003(1.382); 5–10min, *p* = 0.004(1.306)], and cold water significantly increased SBP at up to 20 min of the recovery period [0–5 min, *p* = 0.0306, d = 0.975; 5–10 min, *p* = 0.0226(0.914); 10–15 min, *p* = 0.0402(0.994); 15–20 min, *p* = 0.016(1.163)]. In the older participants, all changes in blood pressure were significantly increased by cold and cold carbonated water at up to 20 min of the recovery period [ΔSBP of cold water in the recovery period at 0–5, 5–10, 10–15, and 15–20 min: *p* = 0.0028(1.71), *p* = 0.0072(1.514), *p* = 0.041(1.127) and *p* = 0.0284(1.065), respectively; ΔMBP of cold water in the recovery period at 0–5, 5–10, 10–15, and 15–20 min: *p* = 0.0016(1.783), *p* = 0.006(1.544), *p* = 0.0138(1.331) and *p* = 0.0106(1.253), respectively; ΔDBP of cold water in the recovery period at 0–5, 5–10, 10–15, and 15–20 min: *p* = 0.004(1.65), *p* = 0.0104(1.38), *p* = 0.0105(1.449) and *p* = 0.0059(1.358), respectively; ΔSBP of cold carbonated water in the recovery period at 0–5, 5–10, 10–15, and 15–20 min: *p* < 0.0001(2.242), *p* = 0.0045(1.405), *p* = 0.011(1.374) and *p* = 0.0024(1.687), respectively; ΔMBP of cold carbonated water in the recovery period at 0–5, 5–10, 10–15, and 15–20 min: *p* < 0.0001(2.368), *p* = 0.0044(1.526), *p* = 0.0023(1.516) and *p* = 0.0006(1.849), respectively; ΔDBP of cold carbonated water in the recovery period at 0–5, 5–10, 10–15, and 15–20 min: *p* < 0.0001(2.266), *p* = 0.0024(1.509), *p* = 0.0044(1.525) and *p* = 0.0009(1.847), respectively]. The ΔSBP from cold and cold carbonated water during the recovery period was about 5–10 mmHg in the younger adults (cold water in the recovery period at 0–5, 5–10, 10–15, 15–20 min: 4.96 ± 3.76, 4.65 ± 3.87, 5.06 ± 3.96, and 5.92 ± 3.17 mmHg, respectively; cold carbonated water in the recovery period at 0–5, 5–10, 10–15, and 15–20 min: 10.75 ± 8.53, 10.16 ± 6.79, 6.46 ± 6.88, and 5.62 ± 7.42 mmHg, respectively), and exceeded 10 mmHg in the older adults (cold water in the recovery period at 0–5, 5–10, 10–15, and 15–20 min: 16.87 ± 7.85, 14.94 ± 11.78, 11.13 ± 11.55, and 10.51 ± 11.1 mmHg, respectively; cold carbonated water in the recovery period at 0–5, 5–10, 10–15, and 15–20 min: 19.64 ± 13.97, 12.31 ± 7.74, 12.04 ± 10.03, and 14.78 ± 12.4 mmHg, respectively). On the other hand, with room temperature water, the pressor response did not outlast the drinking period in either group.

The changes in blood pressure from cold and cold carbonated water were greater in the older than in the younger participants. With cold water, ΔSBP was significantly higher in the older than in the younger adults at the drinking period [*p* = 0.0242(1.233)], ΔSBP and ΔMBP were significantly higher in the older than in the younger adults at 0–5 and 5–10 min of the recovery period [ΔSBP in the recovery period at 0–5 and 5–10 min: *p* = 0.0015(2.069) and *p* = 0.0318(1.282); ΔMBP in the recovery period at 0–5 and 5–10 min: *p* = 0.0009(2.235) and *p* = 0.0069(1.56)], and ΔDBP was significantly higher at all time points during the recovery period [the recovery period at 0–5, 5–10, 10–15, and 15–20 min: *p* = 0.0022(1.899), *p* = 0.0023(1.503), *p* = 0.0384(1.149) and *p* = 0.0443(1.122), respectively]. With cold carbonated water, ΔMBP was significantly higher in the older than in the younger adults at 10–15 and 15–20 min of the recovery period [the recovery period at 10–15 and 15–20 min: *p* = 0.0477(0.985) and *p* = 0.0306(1.127)], and ΔDBP was significantly higher in the older than in the younger adults at all time points during the recovery period [the recovery period at 0–5, 5–10, 10–15, and 15–20 min: *p* = 0.0347(1.025), *p* = 0.0496(0.895), *p* = 0.015(1.226) and *p* = 0.0169(1.233), respectively].

HR was significantly higher HR in the drinking than in the control period in both groups [younger adults, *p* < 0.0001(3.311); older adults, *p* < 0.0001(2.888)], but significantly lower HR during the recovery period [5–10, 10–15, and 15–20 min of the recovery period in younger adults: *p* = 0.008 (0.859), *p* = 0.002(0.752) and *p* = 0.0009(0.83), respectively; 10–15 and 15–20 min of the recovery period in older adults: *p* = 0.02(0.968) and *p* = 0.0092(1.093)].

**Figure 2:**
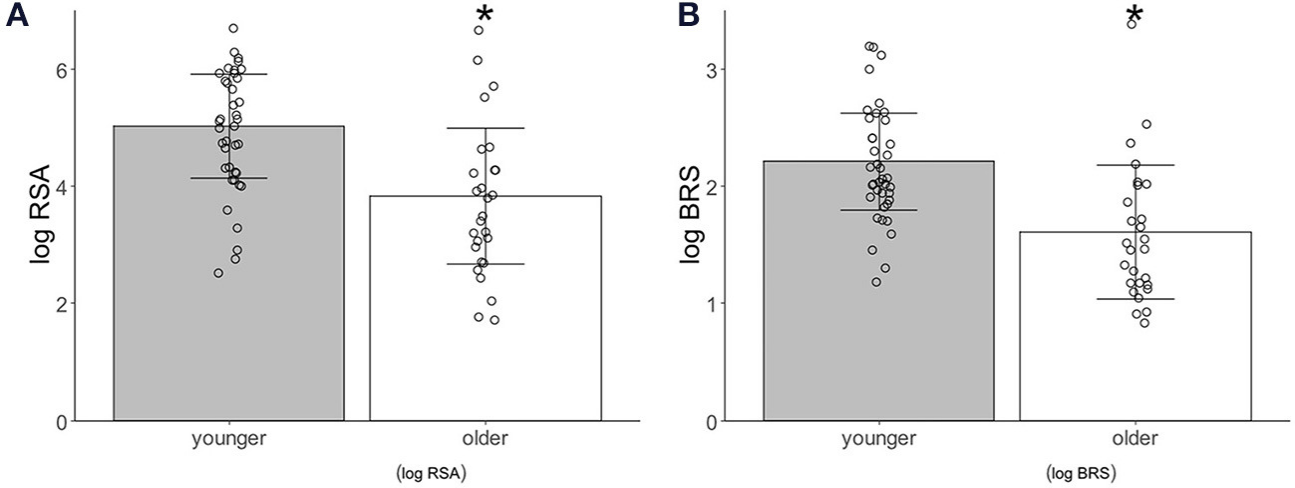
Autonomic activity index associated with age. **(A)** Logarithm-transformed respiratory sinus arrhythmia (logRSA). **(B)** Logarithm-transformed baroreflex sensitivity (logBRS). Values are shown as mean ± standard deviation, *p < 0.05 vs. younger.

The logRSA [younger adults, 5.02 ± 0.88; older adults, 3.82 ± 1.10, *p* = 0.0002(1.061)] and logBRS [younger adults, 2.21 ± 0.41; older adults, 1.65 ± 0.54, *p* < 0.0001(1.118)] during the control period were significantly higher in the younger than in the older participants (Figure 2).

### Core Temperature

**Figure 3:**
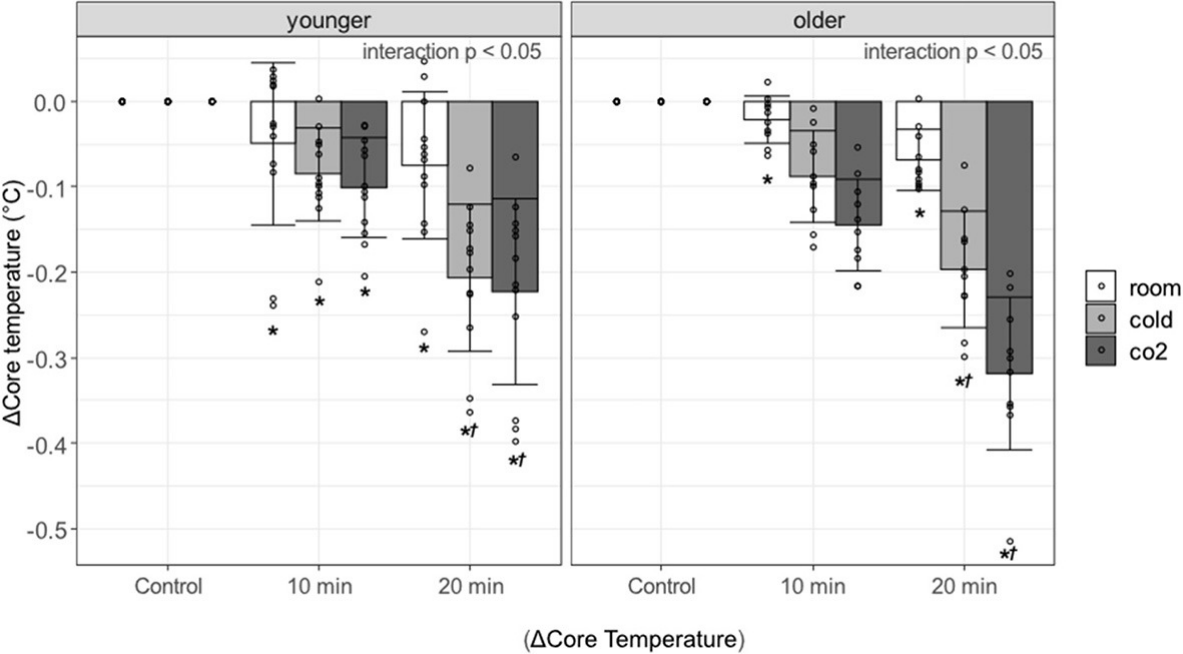
Changes in core temperature against control period, associated with age, type of water, and period. Values are shown as mean ± standard deviation, *p < 0.05 vs. control period, †*p* < 0.05 vs. room.

Figure 3 shows the change in core temperature at 0–10 and 10–20 min during the recovery period and the results of the multiple comparisons. An interaction effect was observed in the younger and older participants (both groups, *p* < 0.0001), so multiple comparisons were performed after examining the effects of each factor. Core temperature was significantly lower during the recovery period as compared with control period in both groups [younger adults in the recovery period at 0–10 and 10–20 min: room water, *p* = 0.0012(1.02) and *p* = 0.0172(1.55), cold water, *p* < 0.0001(1.47) and *p* < 0.0001(3.55), cold carbonated water, *p* < 0.0001(1.74) and *p* < 0.0001(3.83); older adults in the recovery period at 0–10 and 10–20 min: room water, *p* = 0.0472(0.953) and *p* < 0.0001(3.04), cold water, *p* < 0.0001(3.89) and *p* < 0.0001(8.69), cold carbonated water, *p* < 0.0001(6.4) and *p* < 0.0001(14.1)]. The core temperature reduction was larger after cold and cold carbonated than after room temperature water. This observation holds for the whole recovery period in the older and for the late recovery period in the younger group [younger adults in the recovery period at 10–20 min: cold water, *p* < 0.0001(2.278), cold carbonated water, *p* < 0.0001(2.564); older adults in the recovery period at 0–10 and 10–20 min: cold water, *p* = 0.0003(1.98) and *p* < 0.0001(2.36), cold carbonated water, *p* < 0.0001(3.67) and *p* < 0.0001(4.61)].

## Discussion

The present study, which involved drinking 200 mL of water, had three major findings: (1) The pressor response to the drinking of cold and cold carbonated water was greater than that to room temperature water during the drinking period in both younger and older adults, especially that to cold carbonated water; (2) the pressor response to cold and cold carbonated water was persistent during the recovery period, and (3) the pressor response during the drinking and recovery periods was greater in the older than in the younger participants.

### Pressor Response in the Drinking Period

In both the younger and older participants, the pressor response was observed while drinking cold, cold carbonated water, or room temperature water. These results were consistent with those from previous studies (10, 29). As mentioned above, Abe et al. (12) suggested in an experimental study in rats that elevated blood pressure is induced by afferent signals of muscle mechanoreceptors related to water drinking. The same mechanism is considered to have affected the pressor response in the present study.

The pressor response during drinking was greater to cold and cold carbonated water than to room temperature water. It has been shown that as a nociceptive stimulus, a cold stimulus to the skin stimulates free nerve endings, increases sympathetic nerve activity, and causes an increase in blood pressure (30, 31). It is likely that a cold stimulus to the oropharyngeal region from the cold and cold carbonated water may have caused a greater pressor response compared with the room temperature water through a similar mechanism. In addition, the pressor response to cold carbonated water was greater than that to cold water. The oral stimulation produced by carbonated water activates the nociceptive pathways of the trigeminal nerve (15). The nociceptive stimulus increases sympathetic nerve activity and blood pressure (14). When drinking cold carbonated water, in addition to the cold stimulus, the nociception with carbonated water may have caused the pressor response to be more significant than that when drinking cold water.

### Pressor Response in the Recovery Period

After drinking room temperature water, elevated pressure was immediately restored in both the older and younger participants. This result in young adults agrees with those from previous studies (10, 32). On the other hand, it has been reported that the pressor response occurs after drinking water in normal older adults as well as in patients with autonomic failure (6), which is different from the results in older participants in this study. This may be due to low water intake and baroreflex regulation. It was reported that drinking 240 ml water in four autonomic failure patients caused a lesser pressor response than drinking 480 ml water (6). After drinking water, HR decreased by baroreflex adjustment (11), and the present results also showed a decrease in HR in both groups. In the present study, we believe that both the small amount of drinking water and the adjustment by the baroreflex acted to suppress pressor response in the elderly.

In most cases, the drop in blood pressure occurred immediately after drinking the water and stabilized within 10 to 30 sec, but the change in a short time varied considerably among individuals, and differences depending on the type of drinking water could not be confirmed. The baroreflex may function during drinking in healthy young participants (10), and the immediate drop in both groups may result from baroreflex adjustment in the present results. Since detailed results were not obtained on this point, further investigation will be necessary for the future.

The decrease in blood pressure after drinking did not return to the level of the control period in cold water and cold carbonated water, and in addition to the persistent increase in blood pressure, the baroreflex also acted, as mentioned above. Baroreflex sensitivity is reduced in orthostatic pre-syncope (33). Drinking cold water or cold carbonated water for orthostatic pre-syncope may increase blood pressure and improve baroreflex function.

Although we hypothesized that the pressor response while drinking all types of water would disappear immediately after drinking the water in all participants because the amount of water in the present study was too small to affect hemodynamic regulation, the present results of cold water and cold carbonated water do not support the hypothesis. Since these results may be due to the effect of decreasing the core temperature, we investigated the change in core temperature after drinking all types of water. It has been reported that drinking cold water lowers the core temperature (25), and in this study, the core temperature also decreased after water drinking. Cold exposure of the body surface is known to cause a decrease in peripheral blood flow and increased venous return, cardiac output, and blood pressure (26). Furthermore, Frank et al. (34) reported that both plasma noradrenaline and blood pressure increase when the core temperature is lowered by intravenous saline infusion without body surface cooling. In the present study, a decrease in core temperature was induced by water drinking without cooling the body surface. We believe that the pressor response was induced by a mechanism similar to that shown by Frank et al. (34).

### Effects of Aging on the Pressor Response

During the recovery period, the pressor response was greater and more long-lasting in the older participants whereas their logRSA and logBRS were lower as reported previously (18, 27, 35). It has been reported that the pressor response is more significant because of decreased vascular compliance in the aged (36, 37). We believe that decreased vagal modulation, vagal baroreflex sensitivity, and vascular compliance in the elderly caused a more pronounced and sustained pressor response than in younger adults. Although, as mentioned above, the HR decreased during the recovery period, speculating that the baroreflex function was not completely impaired.

Our results were similar for both young males and females, and we do not believe that gender differences fundamentally affect the results in young adults. Gender differences in autonomic regulatory function may occur even in the elderly (38–41). However, from previous studies, gender differences in autonomic regulation in the elderly appear to be part of the age-related changes. Although only females were included in this study, the decline in logBRS and logRSA is consistent with the trend in the elderly, as described above, and reflects the effects of aging.

### Limitations

In the present study, we did not evaluate the effects of water drinking on orthostatic stress. The effects of cold and cold carbonated water on standing load should be investigated in a future study. Carbonated and mineral waters may have different mineral content and, therefore, osmolarity. For closer examination, it may be necessary to adjust the mineral content. To investigate the effect of water temperature strictly, it may be necessary to conduct the experiment in saline water, considering the effect of low osmolarity after drinking water on the hemodynamics. In the process of recruiting participants, we were unable to gather elderly males, resulting in all participants being female. As mentioned above, even if the experiment includes elderly males, the essential trend is not likely to change the present results. However, the effects of gender differences need to be carefully examined. Additional studies with older males are needed in the future.

## Conclusion

We found that drinking 200 mL of room temperature water increased blood pressure (MBP) by about 10 mmHg during drinking, similar to the results of Endo et al. (10), and that switching to cold water or cold carbonated water further increased the pressor response during drinking and maintained it after drinking. In previous studies on water drinking, the amount of water consumed at one time was about 500 mL (1, 2, 6, 9, 10, 32). In the present study, we used less than half of that amount. We believe that drinking about 200 mL of water is within the range of water that people drink daily and can be easily applied to clinical practice. We speculate that drinking cold or cold carbonated water may be an appropriate first aid method for certain forms of acute hypotension. This method may be especially beneficial in older adults because of their more intense pressor response. It is tempting to assume that the triad of low osmolarity, coldness, and carbonation works in an additive manner also in autonomic failure patients. Future studies may investigate if the stronger pressor effect to cold carbonated water in this population is helpful in this condition.

Abe et al. (12) pointed out the risk of cardiovascular events in regard to the pressor response caused by water drinking. In hypertensive patients, cold soda and cold water may increase the risk of adverse events in daily life, so this should be kept in mind.

## Data Availability Statement

The raw data supporting the conclusions of this article will be made available by the authors, without undue reservation.

## Ethics Statement

The studies involving human participants were reviewed and approved by the Ethics Committee of the International University of Health and Welfare. The patients/participants provided their written informed consent to participate in this study.

## Author Contributions

SK and YE conceived, designed the research, analyzed the data and edited, and revised the manuscript. SK, MK, HM, and TS recruited the participants or performed the experiments. All authors read and approved the final manuscript.

## Funding

This work was supported by Grants-in-Aid for Scientific Research from the Japan Society for the Promotion of Science (B Grant No. 16H02893 and C Grant No. 20K10585).

## Conflict of Interest

The authors declare that the research was conducted in the absence of any commercial or financial relationships that could be construed as a potential conflict of interest.

## Publisher's Note

All claims expressed in this article are solely those of the authors and do not necessarily represent those of their affiliated organizations, or those of the publisher, the editors and the reviewers. Any product that may be evaluated in this article, or claim that may be made by its manufacturer, is not guaranteed or endorsed by the publisher.
